# Methods to Improve Lithium Metal Anode for Li-S Batteries

**DOI:** 10.3389/fchem.2019.00827

**Published:** 2019-12-10

**Authors:** Xiaosong Xiong, Wenqi Yan, Chaolin You, Yusong Zhu, Yuhui Chen, Lijun Fu, Yi Zhang, Nengfei Yu, Yuping Wu

**Affiliations:** State Key Laboratory of Materials-Oriented Chemical Engineering, School of Energy Science and Engineering, Nanjing Tech University, Nanjing, China

**Keywords:** lithium-sulfur battery, anode, dendrite, safety, gel polymer electrolyte, coating

## Abstract

The lithium-sulfur (Li-S) battery has received a lot of attention because it is characterized by high theoretical energy density (2,600 Wh/kg) and low cost. Though many works on the “shuttle effect” of polysulfide have been investigated, lithium metal anode is a more challenging problem, which leads to a short life, low coulombic efficiency, and safety issues related to dendrites. As a result, the amelioration of lithium metal anode is an important means to improve the performance of lithium sulfur battery. In this paper, improvement methods on lithium metal anode for lithium sulfur batteries, including adding electrolyte additives, using solid, and/or gel polymer electrolyte, modifying separators, applying a protective coating, and providing host materials for lithium deposition, are mainly reviewed. In addition, some challenging problems, and further promising directions are also pointed out for future research and development of lithium metal for Li-S batteries.

## Introduction

As a kind of lithium metal secondary battery, lithium-sulfur battery is very likely to be another energy storage device for its high theoretical energy density (2,600 Wh kg^−1^) and specific capacity (1,675 mAh g^−1^) (Lu et al., 2013; Tao T. et al., 2017; Li J. H. et al., 2019). Meanwhile, using sulfur as cathodic material makes it cheaper and more environmentally friendly (Yin et al., 2013). However, the shuttle effect and uneven deposition of lithium limit the practical application of lithium-sulfur batteries (Li et al., 2014; Manthiram et al., 2014). Other problems such as poor electrical conductivity and severe volume change of sulfur also limit the performance of batteries to a certain extent (Evers and Nazar, 2013). At present, the improvement of lithium sulfur battery mainly focuses on the cathode, while much less research has been done on lithium metal anodes.

In Li-S batteries, the metallic lithium is oxidized to produce lithium ion firstly. Unfortunately, the stripping of lithium is uneven commonly, which affects the uniform deposition of lithium in the next step. The uneven and porous lithium deposition layer leads to a large change in its volume, fracturing the fragile solid electrolyte interface (SEI) and then consuming the inner fresh lithium to form a new SEI after reacting with the electrolyte. Polysulfides formed during the charging process transfer to the Li metal anode via the electrolyte, react with lithium irreversibly. At the same time, the uneven deposition of lithium leads to the enrichment of lithium ions in the tip region, and aggravating the growth of lithium dendrites. When the dendrite grows to a certain extent, the electrical contact with the substrate will be broken to produce the unreactive “dead” lithium, which increases the internal resistance and attenuates the capacity of the battery. To make matters worse, dendrites can even pierce through separator, posing a safety hazard. These challenges lead to the drawbacks of Li-S batteries.

So, the improvement of lithium metal anode could be classified into two aspects: protecting active lithium from side reactions and guiding uniform deposition of lithium. Since gain/loss of one electron especially that in 2s orbit should be very fast, the solutions to Li metal are not fully addressed, and there are few reviews on Li metal anode (Cheng et al., 2017; Yan et al., 2019). As a result, here we summarized some promising methods: (1) Adding additives to the electrolyte. (2) Using solid electrolytes or gel polymer electrolytes (Marom et al., 2011). (3) Modifying the separators. (4) Coating protective layers on the surface of lithium directly. (5) Providing host materials for lithium deposition. We summarized the above five directions in the recent 5 years; challenges and further directions are also pointed out.

## Adding Additives to the Electrolyte

Organic liquid electrolyte, especially ether-based electrolyte, is commonly used in lithium-sulfur batteries for its high ionic conductivity, good interface contact with electrodes, and less side reaction with lithium. However, issues originating from the dissolution of intermediate polysulfides make it necessary to add suitable additives to protect the lithium metal anode. So far, the additives include nitrates, iodides, sulfur-containing compounds, and various organic compounds, and their functions are listed in Table S1.

**Figure 1:**
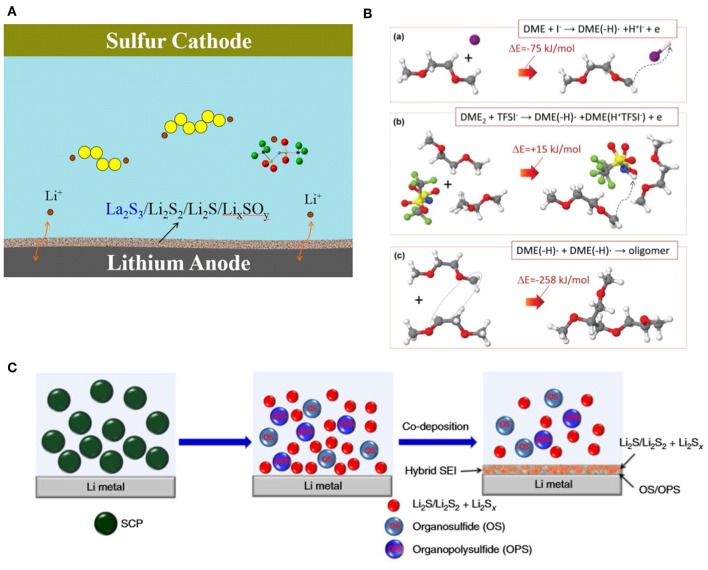
**(A)** Schematic of passivation layer composition when adding La(NO_3_)_3_ additive (Liu S. et al., 2016) Copyright 2016 American Chemical Society. **(B)** Quantum chemistry calculations: formation steps of comb-branched polyether protective film (Wu et al., 2015a). Copyright 2015 John Wiley and Sons. **(C)** Schematic illustration of the SEI layer composition (Li G. et al., 2017). Copyright 2017 Springer Nature.

It is well-known that LiNO_3_ has the ability to protect the lithium metal anode effectively by participating in forming a robust SEI layer *in situ* on the metal surface (Liang et al., 2011). The composition of SEI was investigated by *Operando* X-ray absorption spectroscopy (XAS); results showed that during the initial discharge process, LiNO_3_ reacted with polysulfides to form Li_2_SO_4_, Li_2_SO_3_, and LiNO_2_, which composed a stable SEI on the surface of anode to hamper the side reactions between polysulfides and lithium (Zhang et al., 2018). Other types of nitrates such as La(NO_3_)_3_ and KNO_3_ were also studied in particular. Both the cation and nitrate can participate in improving the stability of SEI on anode (Figure 1A) (Liu S. et al., 2016), and a Li-S full battery using 1 M LiTFSI (DME:DOL = 1:1 v:v) electrolyte added with 0.1 M KNO_3_ exhibited an average discharging capacity of 687 mAh/g within 100 cycles, which was higher than the one with 0.1 M LiNO_3_ (average 637 mAh/g) (Jia et al., 2016).

Iodide, such as LiI and InI_3_, had also acquired outstanding breakthrough (Eo et al., 2009; Liu et al., 2017). Quantum chemistry calculations showed that the I^·^ radicals reacted with DME to form comb-branched polyether in solution (Figure 1B), which suppress the dissolution of polysulfides and then protect the Li metal anode simultaneously (Wu et al., 2015a). Poly(sulfur-random-triallylamine) (PST) (Figure 1C) (Li G. et al., 2017), SOCl_2_ (Li S. et al., 2019), 1,1,2,2-tetrafluoroethyl-2,2,3,3-tetrafluoropropyl ether (TTE) (Qian et al., 2018), and magnesium oxide nanoparticles (Ponraj et al., 2016) were also proven to be effectual.

In order to reduce the polysulfides in electrolyte during the cycling of Li-S battery, additives such as dithiothreitol (DTT), which can slice the S–S bond to accelerate the conversion from polysulfides to Li_2_S_2_/LiS_2_ (Liu M. et al., 2019), biphenyl-4,4′-dithiol (BPD) (Wu H. L. et al., 2017) and carbon disulfide (CS_2_) (Gu et al., 2016), which could react with polysulfides to reduce the chance of contact with the anode, were developed.

## Using Other Types of Electrolyte

Ionic liquid electrolyte is an electrolyte composed entirely of anion and cation, which determines its high safety, excellent stability, and strong solubility to lithium salt (Ghandi, 2014). Moreover, the weak solvation of ionic liquids can reduce the solubility of polysulfides. However, ionic liquids usually have high viscosity, which leads to low ionic conductivity. Adding an appropriate amount of solvents such as DME and DOL to achieve a balance between ionic conductivity and solubility of polysulfides is a suitable choice (Yang et al., 2017). For example, the room temperature ionic conductivity of *N*-methoxyethyl-*N*-methylpyrrolidinium bis (trifluoromethanesulfonyl)-imide (P_1,2O1_TFSI) with 30 wt.% TEGDME as the diluent was increased to 4.303 mS cm^−1^, the Li-S full battery using the electrolyte showed an initial discharge capacity of 1,264 and 911.4 mAh g^−1^ was retained after 50 cycles (Wu et al., 2015b).

Solid electrolyte is also an important direction to improve lithium metal anode by blocking the polysulfides physically. Generally, it can be classified into inorganic solid electrolyte and polymer solid electrolyte. Inorganic solid electrolytes are mainly composed of sulfides, oxides, and phosphates. Examples of sulfides are Li_6_PS_5_Cl (Figure S1A), Li_3_PS_4_, and Li_10_GeP_2_S_12_ (Yamada et al., 2015; Han et al., 2016; Yao et al., 2017). Oxides include Li_3.3_La_0.56_TiO_3_, Li_7_La_3_Zr_2_O_12_, and Li_14_ZnGe_4_O_16_ (Zheng et al., 2014; Yu et al., 2015). Li_1.3_Al_0.3_Ge_1.7_(PO4)_3_, Li_1.3_Al_0.3_Ti_1.7_(PO4)_3_, and Li_2_P_5_O_6_N_5_ (Monchak et al., 2016; Meesala et al., 2017) are typical representatives of phosphates. Polymer solid electrolytes are based on polymer matrix such as PEO, PMMA, PVDF, and PAN without liquid phase. It is characterized by chemical stability, mechanical persistence (durability), and flexibility. However, the low room temperature ionic conductivity plagues the direct application of them (Lin et al., 2016a), and methods such as doping (Monchak et al., 2016), blending, and cross-linking are applied to improve the performance of solid electrolyte. Among them, adding fillers may be the most effective route. Fillers such as LiN_3_ (Eshetu et al., 2017), Li_7_La_3_Zr_2_O_12_ (LLZO) (Figure S1B) (Tao X. et al., 2017), MoS_2_ (Xu et al., 2017), food grade starch (Lin Y. et al., 2016), and halloysite (Lin et al., 2017) were proven to be effectual. Besides, constructing multilayer solid electrolyte by coupling a dense layer that provides supporting function and hosts for electrolytes with a thin layer to block the polysulfides and inhibit the growth of dendrites (Figure S1C) (Fu et al., 2017) was another promising solution.

As an intermediate of liquid electrolyte and all-solid electrolyte, the gel-polymer electrolyte (GPE) therefore has relatively high ionic conductivity and the ability to inhibit polysulfide shuttle (Figure S1D) (Choi et al., 2017). But this GPE is not perfect since its mechanical properties are poor and the ionic conductivity still has room for improvement. The main improvement methods are adding nano-fillers, compounding with plasticizers, and so on (Figure S1E) (Cheng et al., 2018). Specific examples of nano-fillers include ZnO, MgO, Al_2_O_3_, polyethylene, and polystyrene (Kim, 2017; Wu N. et al., 2017; Tripathi and Kumar, 2018).

## Modification on Separators

Coating functional layers that can block polysulfides and modify lithium metal anode on the surface of common separator is also an important way, the actions of which are summarized in Table S2. A layer of porous carbon material on the surface of the separator not only can immobilize polysulfides but also can be used as an upper current collector to improve the utilization rate of active materials. Hence, various carbon materials are used to modified the separator, such as mesoporous carbon (Balach et al., 2015), porous graphene (Zhai et al., 2017), super-P (Zhu et al., 2016), microporous carbon nanofiber (Figure S2A) (Chung et al., 2015), and acetylene black (Figure S2B) (Yang et al., 2019). A multifunctional separator integrated with one or more layers is also a popular improvement direction. Utilizing the synergy effect of carbon and BN on the two sides of a membrane, the performance of the Li-S battery using 1 M LiTFSI in DIOX and DME electrolyte (v:v = 1:1) had been significantly improved; there was still 702 mAh g^−1^ specific capacity at a rate of 4C (Figure S2C) (Kim et al., 2017). On the same principle, a modified separator with compounds of Ketjen Black and MnO coated on Celgard 2400 membrane was developed (Qian et al., 2016).

MXenes are a kind of ultrathin two-dimensional materials that have very high conductivity and surface homogeneity (Figure S2D) (Anasori et al., 2017). By coating Ti_3_C_2_T_X_ (T is -O, -OH, or -F) on commercial Celgard 2400 separator, a superior composite membrane can be obtained (Figure S2E). At a rate of 0.5C, the capacity of the Li-S battery after 500 cycles was 550 mAh g^−1^, and the capacity attenuation per cycle was only 0.063% in an electrolyte of 1 M LiTFSI in DME and DOL (v:v = 1:1) (Song et al., 2016).

## Coating on the Surface of Lithium

Applying a protective layer directly on the surface of the Li anode is also a facile and effective means. The main method is coating some protective layers such as carbon-based materials, polymer, alloy, and ceramic layer, whose actions are summarized in Table S3.

Without question, the protective layer should have sufficient lithium ionic conductivity, chemical inertness, and superior stability. Various carbon materials such as carbon nanofiber (CNF) (Figure S3A) (Zhang A. Y. et al., 2016), nitrogen-doped few-layer graphene (N-FLG) sheets (Figures S3B, S4A) (Kang et al., 2016), artificial graphite particles (Sun et al., 2016), multi-walled carbon nanotube (Deng et al., 2019), and ladderlike carbon nanoarrays (Figure S4B) (Liu L. et al., 2018) were used to homogenize the current density and promote the even deposition of lithium.

Various polymers also provide a wide range of options for surface modification because of the unique characters (Liu Z. C. et al., 2019). For example, Li-S battery using a lithium metal anode modified with a soft, viscous polymer layer showed excellent cycling stability (Figure S3C), with 737 mAh g^−1^ specific capacity after 300 cycles at 0.2C when using an electrolyte of 0.6 M LiTFSI in DOL/DME (v:v, 1/1) with 0.4 M LiNO_3_ as an additive (Zheng et al., 2016). This novel protective layer owned characteristic of slow flow, so it can homogenize the flow of lithium ions and then inhibit dendrite growth (Figure S4C); another advantage is that the process for modifying a polymer protective layer is relatively simple. A 4-μm-thick β-PVDF coating on lithium metal anode made the coulombic efficiency of Cu-Li cells remain 98% in 200 cycles at 1 mA/cm^2^ when using an electrolyte of 1 M LiTFSI in DOL and DME (v:v = 1:1) with 3 wt.% LiNO_3_ additive (Figure S4D) (Luo et al., 2018).

## Providing Host Materials for Lithium Deposition

Layered or three-dimensional frame structure compound has been considered as an effective route to avoid the issues such as the uneven lithium deposition, severe volume change, and safety risks. The frame structure materials include carbon-based materials, polymer materials, metallic materials, and so on, and their actions are summarized in Table S4.

**Figure 2:**
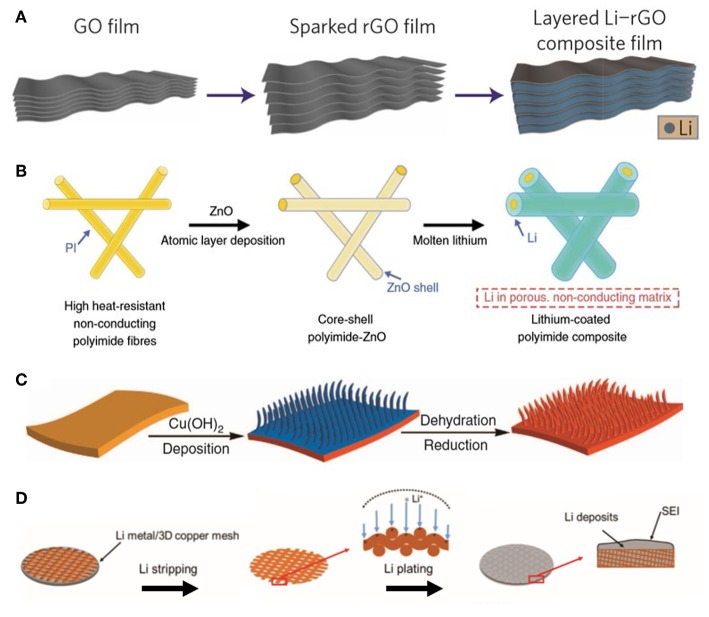
**(A)** Schematic of the preparation process of a layered Li-rGO composite film (Lin et al., 2016b). Copyright 2016 Springer Nature. **(B)** Composite Li anode based on electrospun polyimide PI coated with a layer of lithiophilic ZnO (Liu Z. C. et al., 2019). **(C)** Preparation of a 3D porous copper (Yang et al., 2015). **(D)** The surface of Li anode modified with copper mesh exhibits uniform Li deposition (Li Q. et al., 2017). Copyright 2017 John Wiley and Sons.

Reduced graphene oxide (Figure 2A and Figure S5A) (Lin et al., 2016b) and three-dimensional non-stacked framework graphene (Figure S5B) (Zhang R. et al., 2016) are good illustrations of carbon-based materials. Adding lithiophilic component such as ZnO could further improve the lithiophilicity and reduce the lithium nucleation overpotential (Figure S5C) (Liang et al., 2016).

Highly cross-linked polymeric substrates have abundant pore channels where the Li^+^ can transport rapidly. By electrospinning technique, a polyimide (PI) mesh with a lithiophilic ZnO shell was obtained (Figure 2B), then molten metal lithium was infused into the matrix to prepare a composite Li anode (Figure S5D) (Liu Y. et al., 2016). Copper is well-suited as an anode current collector for its high conductivity and stability at low potential. Hence, numerous studies were focused on 3D Cu; notable examples of it are 3D Cu/Li (Figure 2C and Figure S5F) (Yang et al., 2015) and copper mesh /Li composite anode (Figure 2D and Figure S5E) (Li Q. et al., 2017).

In addition, lithium alloy is also a simple and scalable way to improve the Li-S battery. For example, a Li-Mg (mass ratio: 18.6:81.4) alloy anode was developed. The Mg matrix provided host for the lithium to acquire an even deposition. Also, Mg element participated in the formation of a robust SEI (Kong et al., 2019). Other types of lithium alloys such as Li/Sn (Qiu et al., 2019) and Li/Al (Zhong et al., 2018) had also been proven effective though more studies are still needed.

## Summary

Due to various methods, the performance of lithium-sulfur battery has been greatly improved. However, batteries tested in the experiment are all coin cells, the relatively low current density and the excess amount of lithium underestimate the problems of lithium metal anode to some extent. It is necessary to test the electrochemical performance under the conditions of high current density (>3.0 mA/cm^2^) (Yan et al., 2019) and matching the amount of lithium with the cathodic active materials, so as to meet the needs of practical application.

In addition, problems in lithium-sulfur batteries are various and correlated. Meanwhile, superior comprehensive performance rather than single performance improvement is more urgently needed. Considering that the gel polymer electrolyte has good compatibility with the electrodes, which is favorable for the uniform deposition and stripping of lithium physically. At the same time, the migration of polysulfides can also be suppressed to some extent. More importantly, the gel polymer electrolyte is simple in manufacturing process and low in cost, which is advantageous for mass production. Therefore, we believe that gel electrolyte, which improved by adding fillers, blending, cross-linking and so on, is a very promising direction.

Lastly, the use of more advanced technology to study the mechanism of lithium metal anodes in lithium-sulfur batteries is also of great help in guiding the improvement strategies. All in all, the understanding and researches on the lithium metal anode in lithium-sulfur batteries are still less, and in order to solve the practical application of lithium-sulfur batteries, more work needs to be done.

## Author Contributions

All authors listed have made a substantial, direct and intellectual contribution to the work, and approved it for publication.

### Conflict of Interest

The authors declare that the research was conducted in the absence of any commercial or financial relationships that could be construed as a potential conflict of interest.

## References

[B1] Anasori B. Lukatskaya M. R. Gogotsi Y. (2017). 2D metal carbides and nitrides (MXenes) for energy storage. Nat. Rev. Mater. 2:16098 10.1038/natrevmats.2016.98

[B2] Balach J. Jaumann T. Klose M. Oswald S. Eckert J. Giebeler L. (2015). Functional mesoporous carbon-coated separator for long-life, high-energy lithium-sulfur batteries. Adv. Funct. Mater. 25, 5285–5291. 10.1002/adfm.201502251

[B3] Cheng X.-B. Huang J.-Q. Zhang Q. (2017). Review—li metal anode in working lithium-sulfur batteries. J. Electrochem. Soc. 165, A6058–A6072. 10.1149/2.0111801jes

[B4] Cheng X. L. Pan J. Zhao Y. Liao M. Peng H. S. (2018). Gel polymer electrolytes for electrochemical energy storage. Adv. Energy Mater. 8:1702184 10.1002/aenm.201702184

[B5] Choi S. Song J. Wang C. Park S. Wang G. (2017). Multifunctional free-standing gel polymer electrolyte with carbon nanofiber interlayers for high-performance lithium-sulfur batteries. Chem. Asian J. 12, 1470–1474. 10.1002/asia.201700402 28497555

[B6] Chung S. H. Han P. Singhal R. Kalra V. Manthiram A. (2015). Electrochemically stable rechargeable lithium-sulfur batteries with a microporous carbon nanofiber filter for polysulfide. Adv. Energy Mater. 5:1500738 10.1002/aenm.201500738

[B7] Deng Y. Lu H. Cao Y. Xu B. Hong Q. Cai W. (2019). Multi-walled carbon nanotube interlayers with controllable thicknesses for high-capacity and long-life lithium metal anodes. J. Power Sourc. 412, 170–179. 10.1016/j.jpowsour.2018.11.037

[B8] Eo S. M. Cha E. Kim D. W. (2009). Effect of an inorganic additive on the cycling performances of lithium-ion polymer cells assembled with polymer-coated separators. J. Power Sour. 189, 766–770. 10.1016/j.jpowsour.2008.08.008

[B9] Eshetu G. G. Judez X. Li C. Bondarchuk O. Rodriguez-Martinez L. M. Zhang H.. (2017). Lithium azide as an electrolyte additive for all-solid-state lithium-sulfur batteries. Angew. Chem. Int. Ed. Engl. 56, 15368–15372. 10.1002/anie.201709305 28994228

[B10] Evers S. Nazar L. F. (2013). New approaches for high energy density lithium-sulfur battery cathodes. Acc. Chem. Res. 46, 1135–1143. 10.1021/ar3001348 23054430

[B11] Fu K. Gong Y. Hitz G. T. McOwen D. W. Li Y. Xu S. (2017). Three-dimensional bilayer garnet solid electrolyte based high energy density lithium metal–sulfur batteries. Energy Environ. Sci. 10, 1568–1575. 10.1039/C7EE01004D

[B12] Ghandi K. (2014). A review of ionic liquids, their limits and applications. Green Sustain. Chem. 4, 44–53. 10.4236/gsc.2014.41008

[B13] Gu S. Wen Z. Qian R. Jin J. Wang Q. Wu M.. (2016). Carbon disulfide cosolvent electrolytes for high-performance lithium sulfur batteries. ACS Appl. Mater. Interfaces 8, 34379–34386. 10.1021/acsami.6b11619 27998100

[B14] Han F. Yue J. Fan X. L. Gao T. Luo C. Ma Z.. (2016). High-performance all-solid-state lithium-sulfur battery enabled by a mixed-conductive Li2S nanocomposite. Nano Lett. 16, 4521–4527. 10.1021/acs.nanolett.6b01754 27322663

[B15] Jia W. Fan C. Wang L. Wang Q. Zhao M. Zhou A.. (2016). Extremely Accessible Potassium Nitrate (KNO3) as the highly efficient electrolyte additive in lithium battery. Acs Appl. Mater. Interfaces 8, 15399–15405. 10.1021/acsami.6b03897 27237827

[B16] Kang H. K. Woo S. G. Kim J. H. Yu J. S. Lee S. R. Kim Y. J. (2016). Few-layer graphene island seeding for dendrite-free Li metal electrodes. ACS Appl. Mater. Interfaces 8:26895–26901. 10.1021/acsami.6b09757 27644110

[B17] Kim J. K. (2017). Hybrid gel polymer electrolyte for high-safety lithium-sulfur batteries. Mater. Lett. 187, 40–43. 10.1016/j.matlet.2016.10.069

[B18] Kim P. J. H. Seo J. Fu K. Choi J. Liu Z. M. Kwon J. (2017). Synergistic protective effect of a BN-carbon separator for highly stable lithium sulfur batteries. Npg Asia Mater. 9:e375–e375. 10.1038/am.2017.51

[B19] Kong L. L. Wang L. Ni Z. C. Liu S. Li G. R. Gao X. P. (2019). Lithium–magnesium alloy as a stable anode for lithium–sulfur battery. Adv. Funct. Mater. 29:1808756 10.1002/adfm.201808756

[B20] Li G. Gao Y. He X. Huang Q. Chen S. Kim S. H.. (2017). Organosulfide-plasticized solid-electrolyte interphase layer enables stable lithium metal anodes for long-cycle lithium-sulfur batteries. Nat. Commun. 8:850. 10.1038/s41467-017-00974-x 29021575 PMC5636837

[B21] Li J. H. Liu Z. C. Zhang Q. B. Cheng Y. Zhao B. T. Dai S. G. (2019). Anion and cation substitution in transition-metal oxides nanosheets for high-performance hybrid supercapacitors. Nano Energy 57, 22–33. 10.1016/j.nanoen.2018.12.011

[B22] Li Q. Zhu S. P. Lu Y. Y. (2017). 3D porous Cu current collector/Li-Metal composite anode for stable lithium-metal batteries. Adv. Funct. Mater. 27:1606422 10.1002/adfm.201606422

[B23] Li S. Dai H. Li Y. Lai C. Wang J. Huo F. (2019). Designing Li-protective layer via SOCl2 additive for stabilizing lithium-sulfur battery. Energy Storage Mater. 18, 222–228. 10.1016/j.ensm.2018.09.012

[B24] Li Z. Huang J. Liaw B. Y. Metzler V. Zhang J. B. (2014). A review of lithium deposition in lithium-ion and lithium metal secondary batteries. J. Power Sour. 254, 168–182. 10.1016/j.jpowsour.2013.12.099

[B25] Liang X. Wen Z. Y. Liu Y. Wu M. F. Jin J. Zhang H. (2011). Improved cycling performances of lithium sulfur batteries with LiNO3-modified electrolyte. J. Power Sour. 196, 9839–9843. 10.1016/j.jpowsour.2011.08.027

[B26] Liang Z. Lin D. Zhao J. Lu Z. Liu Y. Liu C.. (2016). Composite lithium metal anode by melt infusion of lithium into a 3D conducting scaffold with lithiophilic coating. Proc. Natl. Acad. Sci. U.S.A. 113, 2862–2867. 10.1073/pnas.1518188113 26929378 PMC4801240

[B27] Lin D. Liu W. Liu Y. Lee H. R. Hsu P. C. Liu K.. (2016a). High ionic conductivity of composite solid polymer electrolyte via in situ synthesis of monodispersed SiO2 nanospheres in Poly(ethylene oxide). Nano Lett. 16, 459–465. 10.1021/acs.nanolett.5b04117 26595277

[B28] Lin D. Liu Y. Liang Z. Lee H. W. Sun J. Wang H.. (2016b). Layered reduced graphene oxide with nanoscale interlayer gaps as a stable host for lithium metal anodes. Nat. Nanotechnol. 11, 626–632. 10.1038/nnano.2016.32 26999479

[B29] Lin Y. Li J. Liu K. Liu Y. X. Liu J. Wang X. M. (2016). Unique starch polymer electrolyte for high capacity all-solid-state lithium sulfur battery. Green Chem. 18, 3796–3803. 10.1039/C6GC00444J

[B30] Lin Y. Wang X. M. Liu J. Miller J. D. (2017). Natural halloysite nano-clay electrolyte for advanced all-solid-state lithium-sulfur batteries. Nano Energy 31, 478–485. 10.1016/j.nanoen.2016.11.045

[B31] Liu L. Yin Y. X. Li J. Y. Guo Y. G. Wan L. J. (2018). Ladderlike carbon nanoarrays on 3D conducting skeletons enable uniform lithium nucleation for stable lithium metal anodes. Chem. Commun. 54, 5330–5333. 10.1039/C8CC02672F 29736511

[B32] Liu M. Chen X. Chen C. Ma T. Huang T. Yu A. (2019). Dithiothreitol as a promising electrolyte additive to suppress the “shuttle effect” by slicing the disulfide bonds of polysulfides in lithium-sulfur batteries. J. Power Sour. 424, 254–260. 10.1016/j.jpowsour.2019.03.113

[B33] Liu M. Ren Y. X. Jiang H. R. Luo C. Kang F. Y. Zhao T. S. (2017). An efficient Li2S-based lithium-ion sulfur battery realized by a bifunctional electrolyte additive. Nano Energy 40, 240–247. 10.1016/j.nanoen.2017.08.017

[B34] Liu S. Li G. R. Gao X. P. (2016). Lanthanum nitrate as electrolyte additive to stabilize the surface morphology of lithium anode for lithium-sulfur battery. ACS Appl. Mater. Interfaces 8, 7783–7789. 10.1021/acsami.5b12231 26981849

[B35] Liu Y. Lin D. Liang Z. Zhao J. Yan K. Cui Y. (2016). Lithium-coated polymeric matrix as a minimum volume-change and dendrite-free lithium metal anode. Nat. Commun. 7:10992. 10.1038/ncomms10992 26987481 PMC4802050

[B36] Liu Z. C. Yuan X. H. Zhang S. S. Wang J. Huang Q. H. Yu N. F. (2019). Three-dimensional ordered porous electrode materials for electrochemical energy storage. Npg Asia Mater. 11:12 10.1038/s41427-019-0112-3

[B37] Lu L. G. Han X. B. Li J. Q. Hua J. F. Ouyang M. G. (2013). A review on the key issues for lithium-ion battery management in electric vehicles. J. Power Sources 226, 272–288. 10.1016/j.jpowsour.2012.10.060

[B38] Luo J. Fang C.-C. Wu N.-L. (2018). High polarity poly(vinylidene difluoride) thin coating for dendrite-free and high-performance lithium metal anodes. Adv. Energy Mater. 8, 1701482-1–1701482-7. 10.1002/aenm.201701482

[B39] Manthiram A. Fu Y. Chung S. H. Zu C. Su Y. S. (2014). Rechargeable lithium-sulfur batteries. Chem. Rev. 114, 11751–11787. 10.1021/cr500062v 25026475

[B40] Marom R. Amalraj S. F. Leifer N. Jacob D. Aurbach D. (2011). A review of advanced and practical lithium battery materials. J. Mater. Chem. 21, 9938–9954. 10.1039/c0jm04225k

[B41] Meesala Y. Jena A. Chang H. Liu R. S. (2017). Recent advancements in Li-ion conductors for all-solid-state li-ion batteries. Acs Energy Lett. 2, 2734–2751. 10.1021/acsenergylett.7b00849

[B42] Monchak M. Hupfer T. Senyshyn A. Boysen H. Chernyshov D. Hansen T.. (2016). Lithium diffusion pathway in Li(1.3)Al(0.3)Ti(1.7)(PO4)3 (LATP) superionic conductor. Inorg. Chem. 55, 2941–2945. 10.1021/acs.inorgchem.5b02821 26930220

[B43] Ponraj R. Kannan A. G. Ahn J. H. Kim D. W. (2016). Improvement of cycling performance of lithium-sulfur batteries by using magnesium oxide as a functional additive for trapping lithium polysulfide. ACS Appl. Mater. Interfaces 8, 4000–4006. 10.1021/acsami.5b11327 26808673

[B44] Qian F. Shao J. Chen Y. Zhu G. Qu Q. Zheng H. (2018). Partially fluorinated ether as an electrolyte additive to modify electrode surface and suppress dissolution of polysulfides in Li-S batteries. Electrochem. Energy Technol. 4, 39–46. 10.1515/eetech-2018-0005

[B45] Qian X. Y. Jin L. Zhao D. Yang X. L. Wang S. W. Shen X. Q. (2016). Ketjen black-MnO composite coated separator for high performance rechargeable lithium-sulfur battery. Electrochim. Acta 192, 346–356. 10.1016/j.electacta.2016.01.225

[B46] Qiu H. Tang T. Asif M. Li W. Zhang T. Hou Y. (2019). Stable lithium metal anode enabled by lithium metal partial alloying. Nano Energy 65:103989 10.1016/j.nanoen.2019.103989

[B47] Song J. Su D. Xie X. Guo X. Bao W. Shao G.. (2016). Immobilizing polysulfides with MXene-functionalized separators for stable lithium-sulfur batteries. ACS Appl. Mater. Interfaces 8, 29427–29433. 10.1021/acsami.6b09027 27723285

[B48] Sun Y. M. Zheng G. Y. Seh Z. W. Liu N. Wang S. Sun J. (2016). Graphite-encapsulated Li-metal hybrid anodes for high-capacity Li batteries. Chem 1, 287–297. 10.1016/j.chempr.2016.07.009

[B49] Tao T. Lu S. Fan Y. Lei W. Huang S. Chen Y. (2017). Anode improvement in rechargeable lithium-sulfur batteries. Adv. Mater. 29:1700542. 10.1002/adma.201700542 28626966

[B50] Tao X. Liu Y. Liu W. Zhou G. Zhao J. Lin D.. (2017). Solid-state Lithium–sulfur batteries operated at 37°C with composites of nanostructured Li7La3Zr2O12/Carbon foam and polymer. Nano Lett. 17, 2967–2972. 10.1021/acs.nanolett.7b00221 28388080

[B51] Tripathi M. Kumar A. (2018). Zinc oxide nanofiller-based composite polymer gel electrolyte for application in EDLCs. Ionics 24, 3155–3165. 10.1007/s11581-018-2504-8

[B52] Wu F. Lee J. T. Nitta N. Kim H. Borodin O. Yushin G. (2015a). Lithium iodide as a promising electrolyte additive for lithium-sulfur batteries: mechanisms of performance enhancement. Adv. Mater. 27, 101–108. 10.1002/adma.201404194 25367318

[B53] Wu F. Zhu Q. Z. Chen R. J. Chen N. Chen Y. Li L. (2015b). A safe electrolyte with counterbalance between the ionic liquid and Tris(ethylene glycol)dimethyl ether for High Performance Lithium-Sulfur Batteries. Electrochim. Acta 184, 356–363. 10.1016/j.electacta.2015.10.109

[B54] Wu H. L. Shin M. Liu Y. M. See K. A. Gewirth A. A. (2017). Thiol-based electrolyte additives for high-performance lithium-sulfur batteries. Nano Energy 32, 50–58. 10.1016/j.nanoen.2016.12.015

[B55] Wu N. Wang W. Wei Y. Li T. H. (2017). Studies on the effect of nano-sized MgO in magnesium-ion conducting gel polymer electrolyte for rechargeable magnesium batteries. Energies 10:1215 10.3390/en10081215

[B56] Xu R. C. Xia X. H. Wang X. L. Xia Y. Tu J. P. (2017). Tailored Li2S-P2S5 glass-ceramic electrolyte by MoS2 doping, possessing high ionic conductivity for all-solid-state lithium-sulfur batteries. J. Mater. Chem. 5, 2829–2834. 10.1039/C6TA10142A

[B57] Yamada T. Ito S. Omoda R. Watanabe T. Aihara Y. Agostini M. (2015). All solid-state lithium-sulfur battery using a glass-type P2S5-Li2S electrolyte: benefits on anode kinetics. J. Electrochem. Soc. 162, A646–A651. 10.1149/2.0441504jes

[B58] Yan C. Zhang X.-Q. Huang J.-Q. Liu Q. Zhang Q. (2019). Lithium-Anode protection in lithium-sulfur batteries. Trends Chem. 1, 693–704. 10.1016/j.trechm.2019.06.007.

[B59] Yang C. P. Yin Y. X. Zhang S. F. Li N. W. Guo Y. G. (2015). Accommodating lithium into 3D current collectors with a submicron skeleton towards long-life lithium metal anodes. Nat. Commun. 6:8058. 10.1038/ncomms9058 26299379 PMC4560781

[B60] Yang D. He L. Liu Y. Yan W. Liang S. Zhu Y. (2019). An acetylene black modified gel polymer electrolyte for high-performance lithium–sulfur batteries. J. Mater. Chem. 7, 13679–13686. 10.1039/C9TA03123E

[B61] Yang Y. B. Men F. Song Z. P. Zhou Y. H. Zhan H. (2017). N-methoxyethyl-N-methylpyrrolidinium bis (trifluoromethanesulfonyl) imide ionic liquid based hybrid electrolyte for lithium sulfur batteries. Electrochim. Acta 256, 37–43. 10.1016/j.electacta.2017.10.020

[B62] Yao X. Y. Huang N. Han F. D. Zhang Q. Wan H. L. Mwizerwa J. P. (2017). High-performance all-solid-state lithium-sulfur batteries enabled by amorphous sulfur-coated reduced graphene oxide cathodes. Adv. Energy Mater. 7:1602923 10.1002/aenm.201602923

[B63] Yin Y. X. Xin S. Guo Y. G. Wan L. J. (2013). Lithium-sulfur batteries: electrochemistry, materials, and prospects. Angew. Chem. Int. Ed. Engl. 52, 13186–13200. 10.1002/anie.201304762 24243546

[B64] Yu S. Schmidt R. D. Garcia-Mendez R. Herbert E. Dudney N. J. Wolfenstine J. B. (2015). Elastic properties of the solid electrolyte Li7La3Zr2O12 (LLZO). Chem. Mater. 28, 197–206. 10.1021/acs.chemmater.5b03854

[B65] Zhai P.-Y. Peng H.-J. Cheng X.-B. Zhu L. Huang J.-Q. Zhu W. (2017). Scaled-up fabrication of porous-graphene-modified separators for high-capacity lithium–sulfur batteries. Energy Storage Mater. 7, 56–63. 10.1016/j.ensm.2016.12.004

[B66] Zhang A. Y. Fang X. Shen C. F. Liu Y. H. Zhou C. W. (2016). A carbon nanofiber network for stable lithium metal anodes with high Coulombic efficiency and long cycle life. Nano Res. 9, 3428–3436. 10.1007/s12274-016-1219-2

[B67] Zhang L. Ling M. Feng J. Mai L. Liu G. Guo J. (2018). The synergetic interaction between LiNO3 and lithium polysulfides for suppressing shuttle effect of lithium-sulfur batteries. Energy Storage Mater. 11, 24–29. 10.1016/j.ensm.2017.09.001

[B68] Zhang R. Cheng X. B. Zhao C. Z. Peng H. J. Shi J. L. Huang J. Q.. (2016). Conductive nanostructured scaffolds render low local current density to inhibit lithium dendrite growth. Adv. Mater. Weinheim. 28, 2155–2162. 10.1002/adma.201504117 26754639

[B69] Zheng G. Y. Wang C. Pei A. Lopez J. Shi F. F. Chen Z. (2016). High-performance lithium metal negative electrode with a soft and flowable polymer coating. Acs Energy Lett. 1, 1247–1255. 10.1021/acsenergylett.6b00456

[B70] Zheng Z. Fang H. Liu Z. Wang Y. (2014). A fundamental stability study for amorphous LiLaTiO3Solid electrolyte. J. Electrochem. Soc. 162, A244–A248. 10.1149/2.0011503jes

[B71] Zhong H. Sang L. Ding F. Song J. X. Mai Y. H. (2018). Conformation of lithium-aluminium alloy interphase-layer on lithium metal anode used for solid state batteries. Electrochim. Acta 277, 268–275. 10.1016/j.electacta.2018.04.191

[B72] Zhu J. D. Ge Y. Q. Kim D. Lu Y. Chen C. Jiang M. J. (2016). A novel separator coated by carbon for achieving exceptional high performance lithium-sulfur batteries. Nano Energy 20, 176–184. 10.1016/j.nanoen.2015.12.022

