# Supplementary material for: Methods to Improve Lithium Metal Anode for Li-S Batteries
Source: Front Chem. 2019 Dec 10;7:827. doi: 10.3389/fchem.2019.00827 (PMC6914760; doi:10.3389/fchem.2019.00827)
Supplement: Supplementary file 1 [file Data_Sheet_1.PDF]

Supporting Information

Methods to improve lithium metal anode for Li-S batteries

Xiaosong Xiong, Wenqi Yan, Chaolin You, Yusong Zhu, Yuhui Chen, Lijun Fu, Yi Zhang, Nengfei Yu*, Yuping Wu*

State Key Laboratory of Materials-oriented Chemical Engineering & School of Energy Science and Engineering, Nanjing Tech University, Nanjing 211816, China
Email: wuyp@fudan.edu.cn

Table S1 |The functions of different electrolyte additives.

Additives	Functions
$LiNO_3$, $La(NO_3)_3$, KNO_3	Forming a protective layer and stable SEI on the surface of the anode.
LiI, InI_3	Suppress the dissolution of polysulfides.<br>Constructing a stable SEI on the anode.
$SOCl_2$, DMDS, CS_2	Inhibiting the shuttle of polysulfides.
Biphenyl-4,4′-dithiol(BPD)	Inhibiting the shuttle of polysulfides.
Dithiothreitol(DTT), Pyrrole	Forming a protective layer on the surface of the anode.

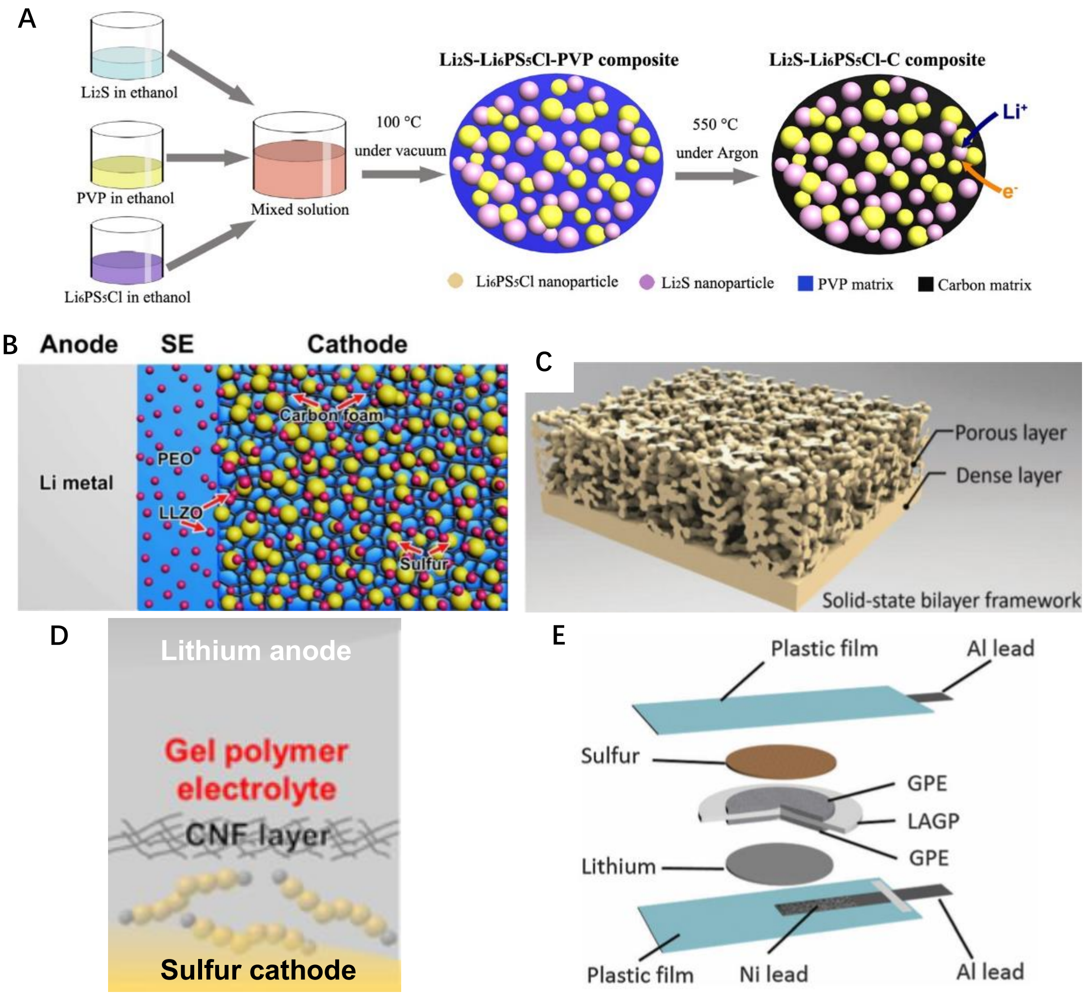

FIGURE S1 | (A) The synthesis process of the conducting Li_2S nanocomposite (Han et al., 2016) Copyright 2016 American Chemical Society. **(B)** an PEO-based solid-state Li−S battery modifying by LLZO nanoparticles (Tao et al., 2017b) Copyright 2017 American Chemical Society. **(C)** solid-state bilayer electrolyte (Fu et al., 2017) Copyright 2017 Royal Society of Chemistry. **(D)** Schematic illustration of the suppression of shuttle effects by GPE/CNF membranes (Choi et al., 2017) Copyright 2017 John Wiley and Sons. **(E)** gel-ceramic multi-layer electrolyte (Wang et al., 2016b) Copyright 2016 Royal Society of Chemistry.

Table S2 | Various separator modification layers and their functions.

Separator modification layer	Examples	Function
Carbon-based materials	porous carbon, graphene, carbon nanotube, carbon nanofiber, super-P, acetylene, ketjen black	Block the polysulfides. Increase the utilization rate of active materials.
Inorganic materials	Black-phosphorous, MoS_2, $BaTiO_3$, Al_2O_3, $Li4Ti_5O_{12}$, SiO_2, BN, $Ti3C_2T_x$,	Block the polysulfides. Increase the ionic conductivity.
Conductive polymer	Nafion, polydopamine	Block the polysulfides.

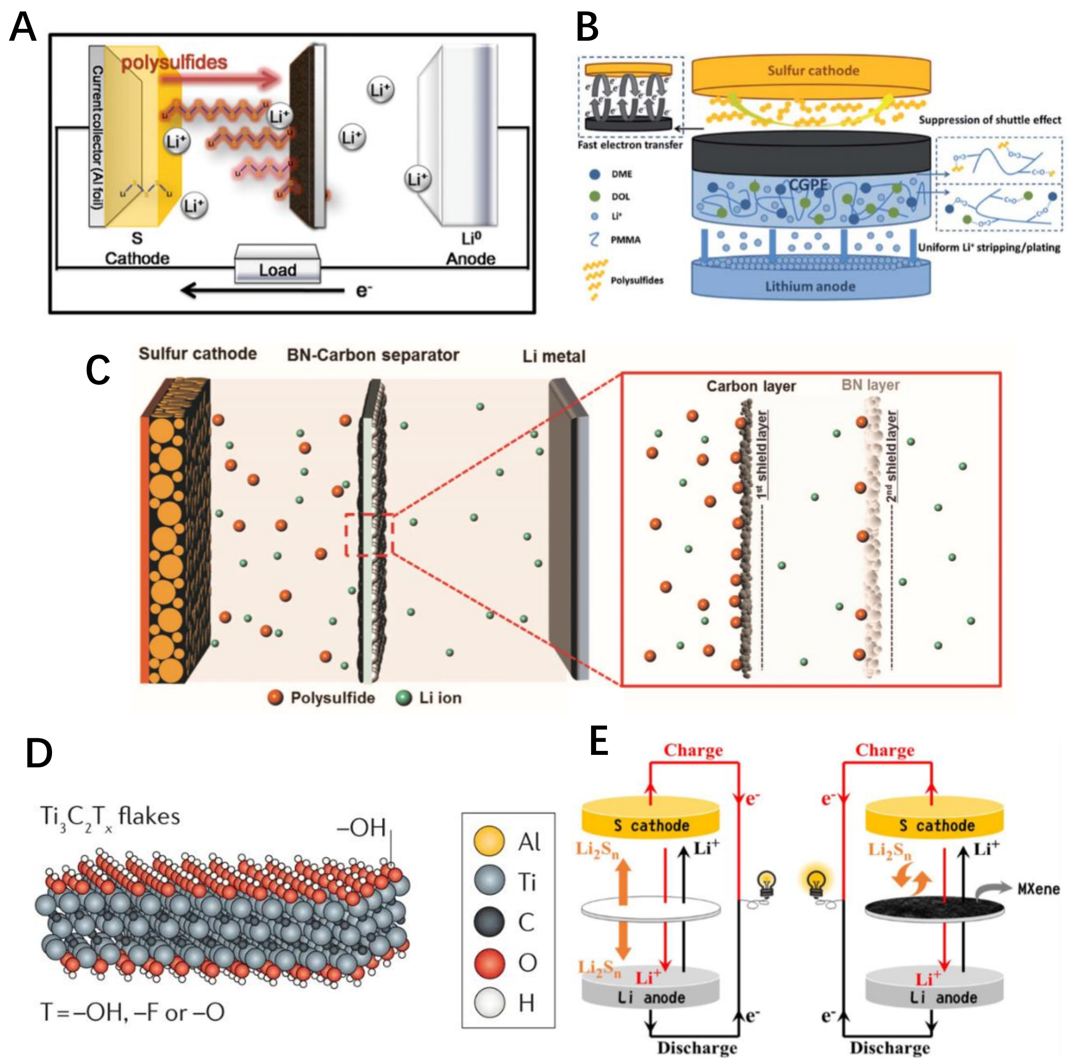

FIGURE S2 | (A) Schematic of ACNF-filter-coated separator (Chung et al., 2015) Copyright 2015 John Wiley and Sons. **(B)** Schematic of a Li–S battery configuration with the CGPE-modified separator (Yang et al., 2019a) Copyright 2019 Royal Society of Chemistry. **(C)** Schematic of a ternary PP/GO/Nafion separator (Kim et al., 2017) Copyright 2017 Springer Nature. **(D)** Schematic of $Ti_3C_4T_x$ flakes (Anasori et al., 2017) Copyright 2017 Springer Nature. **(E)** Illustration of the battery with PP(left) separator and Mxene coated separator(right) (Song et al., 2016) Copyright 2016 American Chemical Society.

Table S3 | Various anode protective layers and their functions.

Protective layer	Example	Functions
Carbon-based layers	Graphite particles, carbon nanospheres, graphene, carbon nanofiber	Homogenize the current density. Block the polysulfides. Inhibit the growth of dendrites
Polymer layer	Poly(dimethylsiloxane), highly viscoelastic polymer	Homogenize the flow of lithium ions. Inhibit the growth of lithium dendrites.
Alloy layer	Li-Al, Li-B, Li-Zn alloy	Reduce the side reaction of lithium. Guide the uniform deposition of lithium.
Others	Al_2O_3, 3D glass fiber	Reduce the volume change of anode. Stable the SEI film.

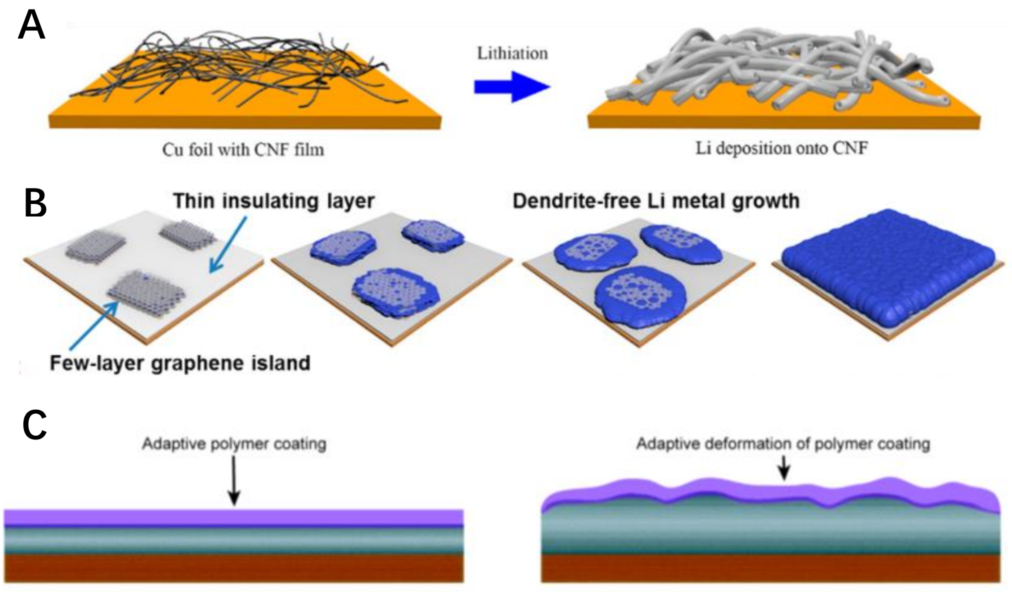

FIGURE S3 | (A) Lithium deposition on CNF Modified copper surface (Zhang et al., 2016a) Copyright 2016 Springer Nature. **(B)** Schematic of Li plating process on few-layer graphene modified anode (Kang et al., 2016) Copyright 2016 American Chemical Society. **(C)** The highly adaptive polymer guides the uniform deposition of lithium (Zheng et al., 2016) Copyright 2016 American Chemical Society.

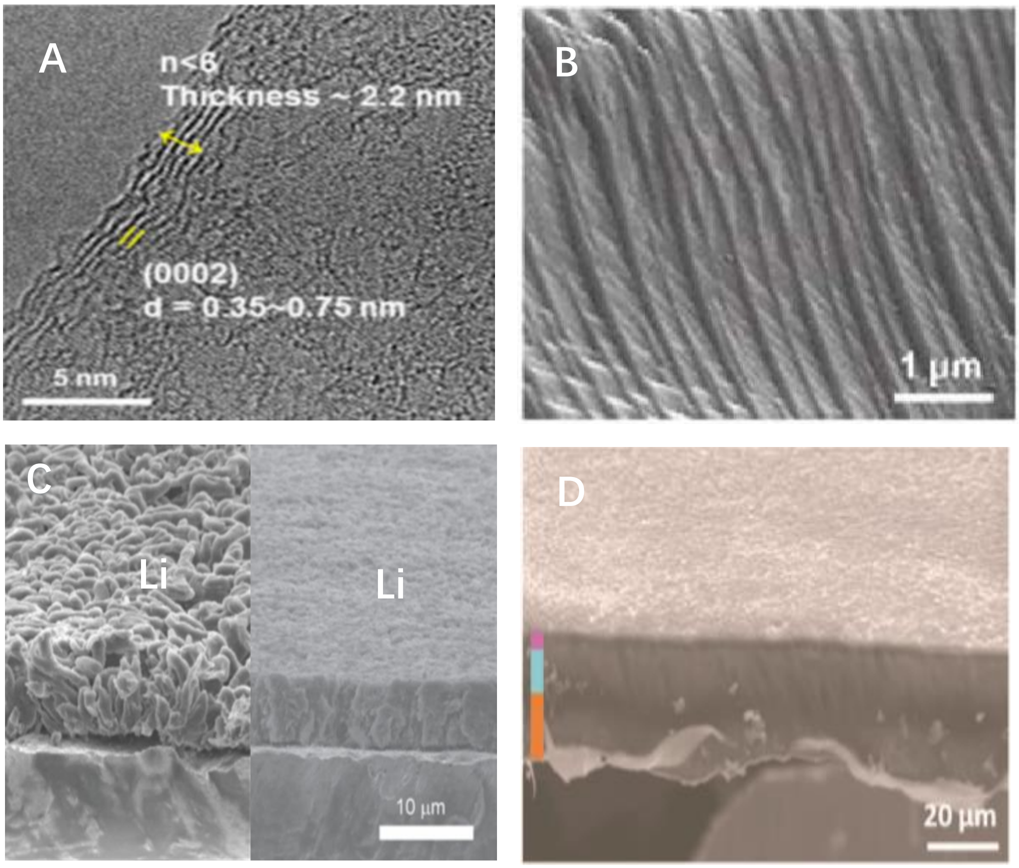

FIGURE S4 | (A) the synthesized N-FLG sheets (Kang et al., 2016) Copyright 2016 American Chemical Society. **(B)** magnified SEM image of ladderlike carbon nanoarrays(Liu et al., 2018) Copyright 2018 Royal Society of Chemistry. **(C)** Lithium Deposition Morphology without (left)/with (right) Polymer Protective Layer (Zheng et al., 2016) Copyright 2016 American Chemical Society. **(D)** Cross section images of Li deposition on β PVDF@Cu with 1 mAh/cm^2 capacity loading at 1 mA/cm^2 (Luo et al., 2018) Copyright 2018 John Wiley and Sons.

Table S4 | Various anode protective layers and their functions.

Framework	Examples	Function
Carbon-based materials	Reduced graphene oxide, porous carbon	Limit the volume change of anode.<br>Guide the uniform deposition of lithium.<br>Stabilize the SEI on anode.
Polymer materials	Polyimide (PI),<br>3D oxidized polyacrylonitrile (PAN) nanofiber	Limit the volume change of anode.<br>Inhibit the growth of lithium dendrites.
Metallic materials	3D porous copper, copper mesh	Limit the volume change of anode.<br>Guide the uniform deposition of lithium.

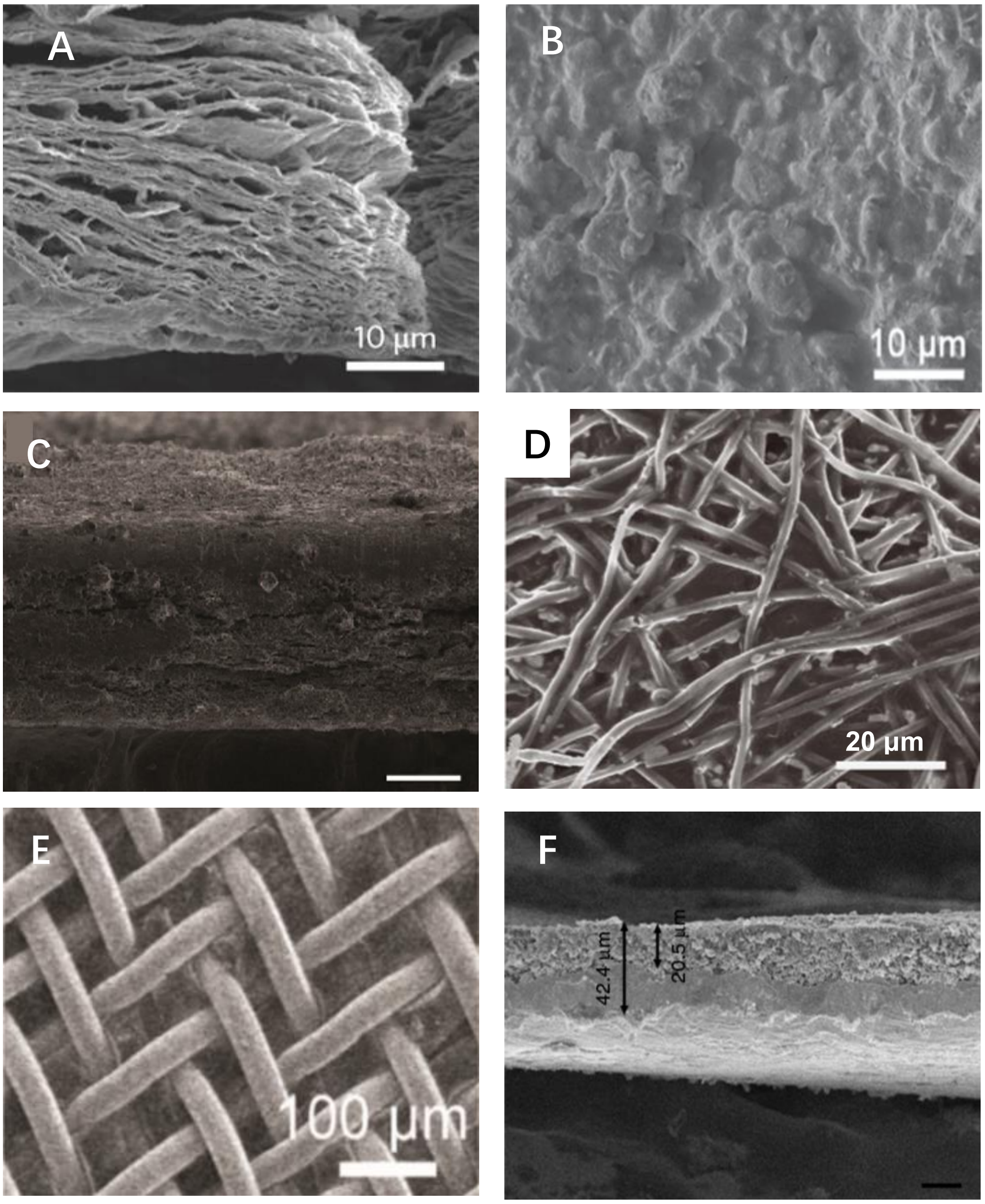

FIGURE S5 | (A) The corresponding SEM images of layered Li–rGO films (Lin et al., 2016b) Copyright 2016 Springer Nature. **(B)** Morphology characterization of graphene-based anode after Li depositing (Zhang et al., 2016b) Copyright 2016 John Wiley and Sons. **(C)** cross-Sectional SEM images of the relatively stable Li/C electrode (Liang et al., 2016) Copyright 2016 National Academy of Sciences. **(D)** SEM image of the porous Li-coated PI matrix (Liu et al., 2016c). **(E)** Surface pictures of the Li-metal/3D copper mesh composite (Li et al., 2017b) Copyright 2017 John Wiley and Sons. **(F)** Cross-section view SEM images of Li deposition on 3D porous Cu (Yang et al., 2015).

References

Han, F.D., Yue, J., Fan, X.L., Gao, T., Luo, C., Ma, Z.H., et al. (2016). High-Performance All-Solid-State Lithium-Sulfur Battery Enabled by a Mixed-Conductive Li2S Nanocomposite. *Nano Letters* 16(7), 4521-4527. doi: 10.1021/acs.nanolett.6b01754.

Tao, X., Liu, Y., Liu, W., Zhou, G., Zhao, J., Lin, D., et al. (2017b). Solid-State Lithium–Sulfur Batteries Operated at 37 °C with Composites of Nanostructured Li7La3Zr2O12/Carbon Foam and Polymer. *Nano Letters* 17(5), 2967-2972. doi: 10.1021/acs.nanolett.7b00221.

Fu, K., Gong, Y., Hitz, G.T., McOwen, D.W., Li, Y., Xu, S., et al. (2017). Three-dimensional bilayer garnet solid electrolyte based high energy density lithium metal–sulfur batteries. *Energy & Environmental Science* 10(7), 1568-1575. doi: 10.1039/c7ee01004d.

Choi, S., Song, J., Wang, C., Park, S., and Wang, G. (2017). Multifunctional Free-Standing Gel Polymer Electrolyte with Carbon Nanofiber Interlayers for High-Performance Lithium-Sulfur Batteries. *Chem Asian J* 12(13), 1470-1474. doi: 10.1002/asia.201700402.

Wang, Q., Wen, Z., Jin, J., Guo, J., Huang, X., Yang, J., et al. (2016b). A gel-ceramic multi-layer electrolyte for long-life lithium sulfur batteries. *Chem Commun (Camb)* 52(8), 1637-1640. doi: 10.1039/c5cc08279j.

Chung, S.H., Han, P., Singhal, R., Kalra, V., and Manthiram, A. (2015). Electrochemically Stable Rechargeable Lithium-Sulfur Batteries with a Microporous Carbon Nanofiber Filter for Polysulfide. *Advanced Energy Materials* 5(18). doi: ARTN 1500738
10.1002/aenm.201500738.

Yang, D., He, L., Liu, Y., Yan, W., Liang, S., Zhu, Y., et al. (2019a). An acetylene black modified gel polymer electrolyte for high-performance lithium–sulfur batteries. *Journal of Materials Chemistry A* 7(22), 13679-13686. doi: 10.1039/c9ta03123e.

Kim, P.J.H., Seo, J., Fu, K., Choi, J., Liu, Z.M., Kwon, J., et al. (2017). Synergistic protective effect of a BN-carbon separator for highly stable lithium sulfur batteries. *Npg Asia Materials* 9(4), e375-e375. doi: ARTN e375.

Anasori, B., Lukatskaya, M.R., and Gogotsi, Y. (2017). 2D metal carbides and nitrides (MXenes) for energy storage. *Nature Reviews Materials* 2(2), 16098. doi: ARTN 16098
10.1038/natrevmats.2016.98.

Song, J., Su, D., Xie, X., Guo, X., Bao, W., Shao, G., et al. (2016). Immobilizing Polysulfides with MXene-Functionalized Separators for Stable Lithium-Sulfur Batteries. *ACS Appl Mater Interfaces* 8(43), 29427-29433. doi: 10.1021/acsami.6b09027.

Zhang, A.Y., Fang, X., Shen, C.F., Liu, Y.H., and Zhou, C.W. (2016a). A carbon nanofiber network for stable lithium metal anodes with high Coulombic efficiency and long cycle life. *Nano Research* 9(11), 3428-3436. doi: 10.1007/s12274-016-1219-2.

Kang, H.K., Woo, S.G., Kim, J.H., Yu, J.S., Lee, S.R., and Kim, Y.J. (2016). Few-Layer Graphene Island Seeding for Dendrite-Free Li Metal Electrodes. *ACS Appl Mater Interfaces* 8(40), 26895-26901. doi: 10.1021/acsami.6b09757.

Zheng, G.Y., Wang, C., Pei, A., Lopez, J., Shi, F.F., Chen, Z., et al. (2016). High-Performance Lithium Metal Negative Electrode with a Soft and Flowable Polymer Coating. *Acs Energy Letters* 1(6), 1247-1255. doi: 10.1021/acsenergylett.6b00456.

Liu, L., Yin, Y.X., Li, J.Y., Guo, Y.G., and Wan, L.J. (2018). Ladderlike carbon nanoarrays on 3D

conducting skeletons enable uniform lithium nucleation for stable lithium metal anodes. *Chem Commun (Camb)* 54(42), 5330-5333. doi: 10.1039/c8cc02672f.

Luo, J., Fang, C.-C., and Wu, N.-L. (2018). High Polarity Poly(vinylidene difluoride) Thin Coating for Dendrite-Free and High-Performance Lithium Metal Anodes. *Advanced Energy Materials* 8(2). doi: 10.1002/aenm.201701482.

Lin, D., Liu, Y., Liang, Z., Lee, H.W., Sun, J., Wang, H., et al. (2016b). Layered reduced graphene oxide with nanoscale interlayer gaps as a stable host for lithium metal anodes. *Nat Nanotechnol* 11(7), 626-632. doi: 10.1038/nnano.2016.32.

Zhang, R., Cheng, X.B., Zhao, C.Z., Peng, H.J., Shi, J.L., Huang, J.Q., et al. (2016b). Conductive Nanostructured Scaffolds Render Low Local Current Density to Inhibit Lithium Dendrite Growth. *Adv Mater* 28(11), 2155-2162. doi: 10.1002/adma.201504117.

Liang, Z., Lin, D., Zhao, J., Lu, Z., Liu, Y., Liu, C., et al. (2016). Composite lithium metal anode by melt infusion of lithium into a 3D conducting scaffold with lithiophilic coating. *Proc Natl Acad Sci U S A* 113(11), 2862-2867. doi: 10.1073/pnas.1518188113.

Liu, Y., Lin, D., Liang, Z., Zhao, J., Yan, K., and Cui, Y. (2016c). Lithium-coated polymeric matrix as a minimum volume-change and dendrite-free lithium metal anode. *Nat Commun* 7, 10992. doi: 10.1038/ncomms10992.

Li, Q., Zhu, S.P., and Lu, Y.Y. (2017b). 3D Porous Cu Current Collector/Li-Metal Composite Anode for Stable Lithium-Metal Batteries. *Advanced Functional Materials* 27(18). doi: ARTN 160642210.1002/adfm.201606422.

Yang, C.P., Yin, Y.X., Zhang, S.F., Li, N.W., and Guo, Y.G. (2015). Accommodating lithium into 3D current collectors with a submicron skeleton towards long-life lithium metal anodes. *Nat Commun* 6, 8058. doi: 10.1038/ncomms9058.
